# Letter Position Coding Across Modalities: The Case of Braille Readers

**DOI:** 10.1371/journal.pone.0045636

**Published:** 2012-10-02

**Authors:** Manuel Perea, Cristina García-Chamorro, Miguel Martín-Suesta, Pablo Gómez

**Affiliations:** 1 ERI-Lectura and Departamento de Metodología, Universitat de València, Valencia, Spain; 2 Organización Nacional de Ciegos (ONCE), Valencia, Spain; 3 DePaul University, Chicago, Illinois, United States of America; University of Leicester, United Kingdom

## Abstract

**Background:**

The question of how the brain encodes letter position in written words has attracted increasing attention in recent years. A number of models have recently been proposed to accommodate the fact that transposed-letter stimuli like jugde or caniso are perceptually very close to their base words.

**Methodology:**

Here we examined how letter position coding is attained in the tactile modality via Braille reading. The idea is that Braille word recognition may provide more serial processing than the visual modality, and this may produce differences in the input coding schemes employed to encode letters in written words. To that end, we conducted a lexical decision experiment with adult Braille readers in which the pseudowords were created by transposing/replacing two letters.

**Principal Findings:**

We found a word-frequency effect for words. In addition, unlike parallel experiments in the visual modality, we failed to find any clear signs of transposed-letter confusability effects. This dissociation highlights the differences between modalities.

**Conclusions:**

The present data argue against models of letter position coding that assume that transposed-letter effects (in the visual modality) occur at a relatively late, abstract locus.

## Introduction

An issue that has attracted increasing attention in recent years is how the brain encodes letters in a written word (see [1] for a review; see also [2] for early evidence). The orthographic coding scheme must be quite flexible because pseudowords generated by transposing letter of words, like jugde (from judge) and caniso (from casino), are frequently classified as words in word/nonword discrimination tasks (lexical decision; see [3]) and, when classified correctly, their response times are substantially longer than those of orthographic controls like jupte or caviro ([4], [5]; see [6], [7] for evidence during normal reading; see [8] for evidence with a rapid serial visual presentation).

To accommodate the presence of transposed-letter effects, a number of researchers have proposed flexible orthographic coding schemes in written-word recognition. While some researchers advocate that the transposed-letter effect is caused by perceptual uncertainty regarding letter position in the visual system (overlap model: [9]; spatial coding model: [10]; noisy Bayesian Reader model: [11]; LTRS model: [12]), other researchers assume that the effect occurs at a more abstract level (at the so-called “visual-word form area”; e.g., open bigram model, [13]; LCD model: [14]), and finally, other researchers have proposed hybrid accounts (e.g., overlap open-bigram model: [15]).

To help determine the locus (and generality) of letter position coding with written words, here we examined letter position coding in another modality (namely, tactile), using Braille. Braille is a system used by visually impaired readers. Each letter is made up of raised dots in a 3×2 matrix. For instance, the transposed-letter pseudowords “caniso” would be 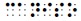 in unabbreviated Braille – in the US the use of abbreviations is common (Grade 2 Braille). Unlike letters in visually presented words, which are processed in a parallel manner ([16]), letters embedded in Braille words are processed sequentially in a left-to-right manner –one letter at a time as the fingers scan through the paper or display ([17], [18]).

The aim of the present study was to examine the flexibility of the input coding scheme for written-word recognition in the tactile modality. Obviously, current models of visual-word recognition implicitly or explicitly assume that the visual system imposes constrains in the word-identification process. Some models explicitly state that transposed-letter effects are due to some perceptual noise not much different from any other binding of location and identity of objects in the visual system (e.g., overlap model or LTRS model). In contrast, other models (namely, open-bigram models like SERIOL and LCD models) posit that transposed-letter effects relate to a level of representation that is more abstract than the retinotopic location of letters (the so-called “visual word form area” see Figure 1 in [14]) –note that Reich, Szwed, Cohen, and Amedi [19] reported fMRI evidence that congenitally blind individuals activate the same “word form area” as sighted individuals. While none of the models have been extended to Braille reading (and we hope that the present work sparks interest in doing so), one can make the prediction that location uncertainty should be drastically reduced in the tactile modality for those models that assume that such uncertainty relates to retinotopic location. Thus, transposed-letter experiments on Braille can help to adjudicate between accounts that attribute letter position coding to either early visual encoding or to later stages of word recognition that occur in the “visual word form area”.

**Figure 1:**
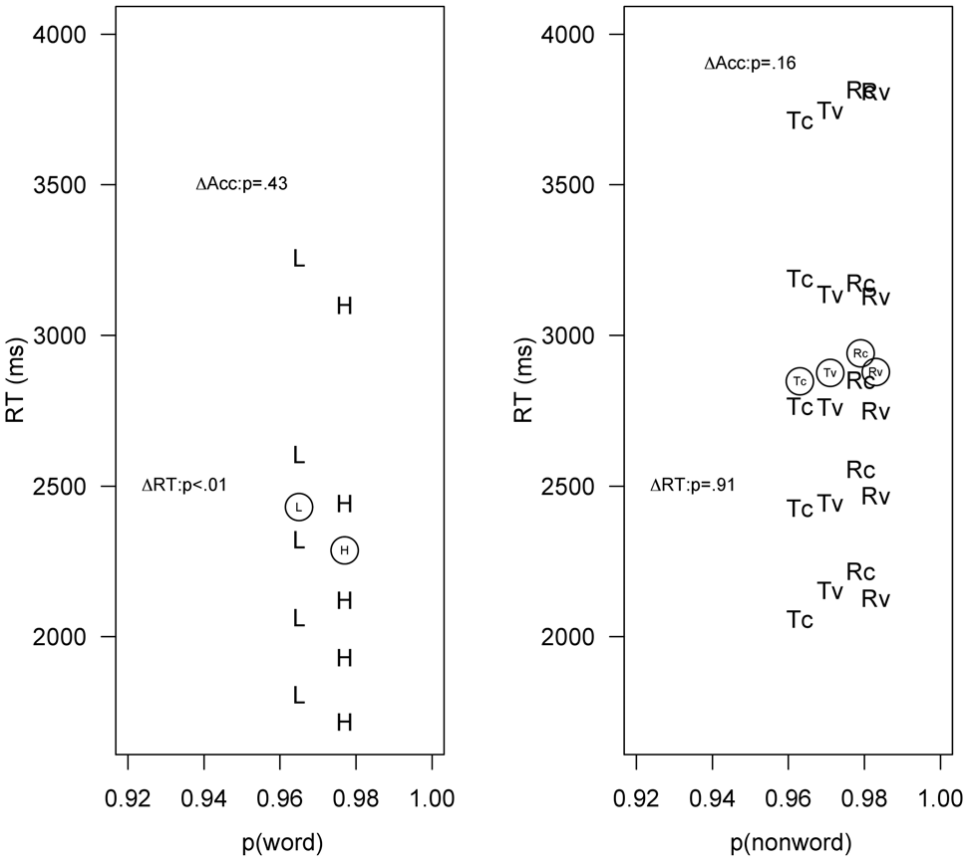
The figure shows the accuracy (in x-axis) and the RTs at the.1,.3,.5,.7 and.9 vincentiles for correct responses to words (H: high frequency, L: low frequency) and pseudowords (Rc: replacement-letter consonant, Tc: transposed-letter consonant; Rv: replacement-letter vowel, Tv: transposed-letter vowel). The circles represent the means of each condition.

We employed a single-presentation lexical decision task similar to that used in the visual modality by Perea and Lupker ([5]; see also [20], [21]). The rationale is the following: If transposed-letter pseudowords activate their corresponding word-unit representations to a higher degree than the appropriate orthographic controls (i.e., replacement-letter pseudowords), one would expect longer decision times and a higher proportion of “word” responses for transposed-letter pseudowords than for the replacement-letter pseudowords (see [5], [20], [21]). The set of pseudowords employed in the present experiment was taken from Carreiras, Perea and Vergara [22], who reported the usual transposed-letter effect: 1032 ms and 24.6% of errors for transposed-letter pseudowords vs. 914 ms and 6.4% for replacement-letter pseudowords. Finally, we should note that in the Perea and Lupker [5] and in the Carreiras et al. [22] experiments, the transposed-letter confusability effect was similar in magnitude in the latency data for consonant transpositions than for vowel transpositions, but it was greater for consonant transpositions in the error data.

In sum, if the locus of letter transposition effects is at an abstract level of processing (i.e., a “multisensory integration area”; see Reich et al. [19]) that may be common to printed and tactile modalities (e.g., the bank of “local bigrams” hypothesized in the occipito-temporal sulcus in the LCD model), then the transposed-letter confusability effect should still be sizeable in the tactile modality. In contrast, if the locus of letter transposition effects is at a retinotopical level (as assumed in the overlap model), the transposed-letter confusability effect should be small or negligible.

## Results

Given that response times in Braille are often over 2 sec ([17]), we present the RT distributions of correct responses ([23]) in addition to the averages (see Figure 1). Very long latencies (over 5 sec; less than 4% of the data) were excluded from the analyses. For words, we examined the effect of word-frequency (low vs. high) on mean correct response times and percent errors. For pseudowords, we examined the effects of Type of pseudoword (transposition vs. replacement) and Type of transposition/replacement (consonants vs. vowels) on mean correct response times and percent errors. The statistical analyses were conducted over participants (F1) and items (F2).

### Word Data

The statistical analyses on the latency data for words revealed a word-frequency effect (144 ms; F1(1,7) = 56.5, p<.001, F2(1,118) = 7.88, p<.007). Indeed, an inspection to the data revealed that all participants showed an advantage for high-frequency over low-frequency words. This implies that, as occurs in visual-word recognition, lexical factors play an important role during lexical access in Braille.

### Pseudoword Data

Unlike the parallel experiment in the visual modality (see above), the data did not revealed any signs of longer response times for transposed-letter pseudowords than for the replacement-letter pseudowords in Braille. Indeed, the effect was (if anything) facilitative rather than inhibitory (see Figure 1), although the difference did not approach significance (both Fs<1). Neither the effect of type of pseudoword nor the interaction between the two factors approached significance, all Fs<1.

The error data only revealed a small (0.7%), nonsignificant, interference effect for the transposed-letter pseudowords relative to the replacement-letter pseudowords (2.9 vs. 2.2% of errors), F1(1,7) = 2.27, p = .18, F2(1,119) = 1.97, p = .16. Neither the effect of type of pseudoword nor the interaction between the two factors approached significance, all Fs<1. Given that ANOVAs may not be the most appropriate procedure to analyze binomially distributed categorical data (e.g., error responses in two-choice experiments; see [24], [25]), the error data for the pseudowords were also analyzed using a series of linear mixed effects models of diminishing complexity of random effects structure (lme4 package in R). The optimal model was the one that had participants and items as random slopes (see [24]). The Laplace approximation (via z values) was employed to fit the binomial data. The analyses revealed that none of the effects approached significance: type of pseudoword: z = −0.925, β = .−0.625, p = .35; type of transposition/replacement, z = .291, β = 0.237, p = .77; interaction effect: z = 0.045, β = 0.035, p = .97.

## Discussion

This is the first study that has examined how letter position coding is achieved in reading using a tactile writing system. As expected, a robust word-frequency effect was obtained for words (see also [17] for similar evidence in a semantic categorization task), reflecting the influence of lexical factors on the process of lexical access in Braille. More important, unlike the parallel experiments with the visual modality in which there was a substantial transposed-letter effect both in RTs and error rates (e.g., an average of 1032 ms and 24.6% of errors for transposed-letter pseudowords vs. 914 ms and 6.4% for replacement-letter pseudowords, in the Carreiras et al. [22] experiment), we found no evidence of an interference effect of transposed-letter confusability in Braille – note that this was the case for both consonant and vowel transpositions. There was only a very small (0.7%) nonsignificant interference effect in the error rates, which was dramatically smaller than that obtained in the visual modality (see [5], [20], [21]). Furthermore, the latency data revealed, if anything, some small facilitation –in particular for the consonant transpositions (see Figure 1). Thus, the obtained pattern of transposed-letter effects in the tactile modality is noticeably different from those in the visual modality, and that is so even if the 0.7% effect in the error rates were significant with a substantially larger sample size.

The differences in results between the tactile and the visual modalities highlight the differences in word processing across modalities. In visually presented words, the information about the location of the letters is not particularly reliable, and this might be because there is perceptual noise in the processing of letter position –consistent with object processing in models of visual attention ([9], [10], [11], [12]). When processing written words in Braille, there is an inherent serial process given that (because of the limitations of the tactile system), only one letter is scanned at a time. The serial processes involved in Braille word recognition may lead to a “slot” input coding scheme that is rather insensitive to transposed-letter effects. Importantly, in a recent article, Reich et al. [19] argued that the “visual word form area” is also recruited during Braille reading, reflecting anatomical and functional consistency between sighted and blind readers. If, as Reich et al. claim, there is significant overlap at the letter/word level regardless of reading modality, then the transposition effects which are found in the visual modality are (probably) not related to letter-specific abstract levels of representation (like abstract “letter bigrams”) but instead, they are related to an earlier visual level (see [9]). Furthermore, as suggested by a reviewer, the comparison between the visual and tactile modalities may also provide an elegant test scenario for models that propose a serial manner of orthographic processing versus those postulating parallel access to orthographic representations.

We believe that further research should be conducted to explore the subtleties of the input coding schemes across modalities and to develop a complete model of Braille written-word recognition and reading. Informal conversations with the participants after the experiment revealed a potentially interesting issue. In experiments using the visual modality, participants typically report that they initially process transposed-letter pseudowords like CHOLOCATE as if they were the real word (i.e., CHOCOLATE). That is, in the visual modality, participants have to make an effort in order not to be able to “reconstruct” the pseudoword as the real word. In contrast, in the present Braille experiment, participants indicated that they could easily notice that pseudowords (transposed-letter pseudowords and replacement-letter pseudowords) were actually pseudowords –indeed error rates were very low– but they also indicated that, with some effort, they were able to volitionally reconstruct the base word of a number of pseudowords.

In sum, the present lexical decision experiment has revealed that the robust transposed-letter effects that occur with isolated pseudoword presentations in the visual modality (e.g., RELOVUTION; see [5], [21], [22]) is absent (or, at least, dramatically diminished) when the experiment is conducted in the tactile modality with Braille readers. This dissociation between modalities argues against models of letter position coding that assume that such transposed-letter effects (in the visual modality) occur at a relatively late, abstract locus. Future research should focus on how context may modulate the process of letter position coding in Braille (e.g., see [26] for evidence of the role of context in letter position coding during normal reading), and also whether the serial processed involved in Braille are shared in another modality which also implies seriality, as in the case of auditory presented words/pseudowords. Finally, more research on Braille reading will contribute to Braille literacy (including the potential reading difficulties in blind children), as Braille fluency has a dramatic impact on the employment and income of blind individuals [27].

## Materials and Methods

### Ethics Statement

All participants gave written informed consent – the experiment was conducted with the approval of the “Comité Ético de Investigación en Humanos de la Comisión de Ética en Investigación Experimental de la Universitat de València” (Ethics Committee for Human Research at the University of Valencia) and the “Organización Nacional de Ciegos de España” [ONCE] (National Organization of Spanish Blind People.).

### Participants

Eight proficient blind Braille readers, all of them university graduates who started learning Braille at age 5, participated in the experiment (M = 35 years; range: 25–49). They received a small monetary compensation (4 €). They were native speakers of Spanish and were naïve as to the purpose of the experiment.

### Apparatus

To present the stimuli and record the responses, we employed a *Freedom Scientific (Focus40)* Braille display connected to a Windows-based computer.

### Materials and Design

For the set of words, we selected 60 words of high-frequency (mean = 114 occurrences/million [range: 37.8–341.4] in the B-Pal database [28], mean length = 8.9 letters [range: 7–11]) and 60 words of low-frequency (mean = 5 occurrences/million [range: 3.9–6.4], mean length = 8.9 letters [range: 7–11]). For the set of pseudowords, we employed 120 pseudowords taken from the Carreiras et al. [22] lexical decision experiment. These pseudowords had been created by transposing/replacing two nonadjacent consonants/vowels in words not employed in the experiment (mean length: 8.9 letters [range: 7–11]; mean frequency of the base words: 23 per million [range: 6–136]): i) transposition of two nonadjacent consonants (TL-consonant pseudoword; e.g., cholocate), ii) transposition of two nonadjacent vowels (TL-vowel pseudoword; e.g., chocalote), iii) replacement of two nonadjacent consonants (the same as in the TL-Consonant condition) (RL-consonant pseudoword; e.g., chotonate), and iv) replacement of two nonadjacent vowels (the same as in the TL-Consonant condition) (RL-vowel pseudoword; e.g., chocilote). The orthographic uniqueness point (i.e., the letter in which no other words are shared in a left-to-right sequence; see Bertelson et al. [17]) was similar for transposed-letter pseudowords and replacement-letter pseudowords (5.2 and 5.3, respectively). Four lists of materials were created to counterbalance the pseudoword stimuli (e.g., if cholocate is in List 1, chotonate would be in List 2, chotonate in List 3, and chocilote in List 4). Stimulus presentation was randomized for each participant.

### Procedure

The experiment took place individually in a silent room. On each trial, the word/pseudoword appeared on the left side of the Braille display until the participant’s response. Participants employed their preferred reading hand on the Braille display. They were instructed to press, with their other hand, the “yes” button if the stimulus was a Spanish word and the “no” button if the stimulus was not a word. Response times were measures from the onset of target presentation. Participants were instructed to make this decision rapidly, trying not to make too many errors – the instructions were exactly the same as in the Carreiras et al. [22] experiment. The inter-stimulus interval was 2.5 sec. The order of trials was randomized for each participant. Twelve practice trials (with the same manipulation as in the experimental trials) preceded the 240 experimental trials. The whole session took around 25–30 min.
